# Novel insights into neuroinflammation: bacterial lipopolysaccharide, tumor necrosis factor α, and *Ureaplasma* species differentially modulate atypical chemokine receptor 3 responses in human brain microvascular endothelial cells

**DOI:** 10.1186/s12974-018-1170-0

**Published:** 2018-05-23

**Authors:** Christine Silwedel, Christian P. Speer, Axel Haarmann, Markus Fehrholz, Heike Claus, Mathias Buttmann, Kirsten Glaser

**Affiliations:** 1 University Children’s Hospital, University of Wuerzburg, Josef-Schneider-Str. 2, 97080 Wuerzburg, Germany; 2 Department of Neurology, University of Wuerzburg, Josef-Schneider-Str. 11, 97080 Wuerzburg, Germany; 3 Institute for Hygiene and Microbiology, University of Wuerzburg, Josef-Schneider-Str. 2, 97080 Wuerzburg, Germany; 4 Department of Neurology, Caritas Hospital, Uhlandstr. 7, 97980 Bad Mergentheim, Germany

**Keywords:** Atypical chemokine receptor 3, Human brain microvascular endothelial cells, Meningitis, Neuroinflammation, *Ureaplasma* species

## Abstract

**Background:**

Atypical chemokine receptor 3 (ACKR3, synonym CXCR7) is increasingly considered relevant in neuroinflammatory conditions, in which its upregulation contributes to compromised endothelial barrier function and may ultimately allow inflammatory brain injury. While an impact of ACKR3 has been recognized in several neurological autoimmune diseases, neuroinflammation may also result from infectious agents, including *Ureaplasma* species (spp.). Although commonly regarded as commensals of the adult urogenital tract, *Ureaplasma* spp. may cause invasive infections in immunocompromised adults as well as in neonates and appear to be relevant pathogens in neonatal meningitis. Nonetheless, clinical and in vitro data on *Ureaplasma*-induced inflammation are scarce.

**Methods:**

We established a cell culture model of *Ureaplasma* meningitis, aiming to analyze ACKR3 variances as a possible pathomechanism in *Ureaplasma*-associated neuroinflammation. Non-immortalized human brain microvascular endothelial cells (HBMEC) were exposed to bacterial lipopolysaccharide (LPS) or tumor necrosis factor-α (TNF-α), and native as well as LPS-primed HBMEC were cultured with *Ureaplasma urealyticum* serovar 8 (Uu8) and *U. parvum* serovar 3 (Up3). ACKR3 responses were assessed via qRT-PCR, RNA sequencing, flow cytometry, and immunocytochemistry.

**Results:**

LPS, TNF-α, and *Ureaplasma* spp. influenced ACKR3 expression in HBMEC. LPS and TNF-α significantly induced ACKR3 mRNA expression (*p* < 0.001, vs. control), whereas *Ureaplasma* spp. enhanced ACKR3 protein expression in HBMEC (*p* < 0.01, vs. broth control). Co-stimulation with LPS and either *Ureaplasma* isolate intensified ACKR3 responses (*p* < 0.05, vs. LPS). Furthermore, stimulation wielded a differential influence on the receptor’s ligands.

**Conclusions:**

We introduce an in vitro model of *Ureaplasma* meningitis. We are able to demonstrate a pro-inflammatory capacity of *Ureaplasma* spp. in native and, even more so, in LPS-primed HBMEC, underlining their clinical relevance particularly in a setting of co-infection. Furthermore, our data may indicate a novel role for ACKR3, with an impact not limited to auto-inflammatory diseases, but extending to infection-related neuroinflammation as well. AKCR3-induced blood-brain barrier breakdown might constitute a potential common pathomechanism.

**Electronic supplementary material:**

The online version of this article (10.1186/s12974-018-1170-0) contains supplementary material, which is available to authorized users.

## Background

The two human *Ureaplasma* species (spp.) *Ureaplasma (U.) urealyticum* and *U. parvum* are among the smallest self-replicating pathogens and, unlike other bacteria, lack a cell wall [1]. They are typical commensals of the adult urogenital tract and are generally considered as being of low virulence [1].

However, due to high colonization rates, difficult cultural detection, and limited clinical and in vitro data, the clinical relevance of *Ureaplasma* spp. remains controversial and may be much more pronounced than contemplated to date [2]. Considered a significant co-factor in chorioamnionitis and premature birth [3–8], *Ureaplasma* spp. are also known to cause severe invasive infections in immunocompromised adults [9–11] as well as pneumonia, sepsis, and meningitis in preterm and term neonates [12–15].

Meningitis and neuroinflammation profoundly contribute to neonatal morbidity and mortality [16]. Long-term sequelae develop in up to 25–50% and comprise cerebral palsy, hydrocephalus, or neurodevelopmental impairment [17, 18]. While the typical pathogens causing neonatal meningitis, such as *Escherichia (E.) coli* and group B *Streptococcus* [19], are well described, little is known about *Ureaplasma*-driven inflammation of the neonatal central nervous system (CNS). Although *Ureaplasma* spp. can be commonly detected in the cerebrospinal fluid of preterm neonates [20, 21], clinical data on *Ureaplasma*-induced meningitis are scarce [14, 15], and in vitro data are non-existent so far. We therefore aimed to establish a cell culture model of *Ureaplasma* meningitis to assess the inflammatory pathomechanisms involved.

Cytokines and their subgroup chemokines act as key regulators in inflammation and immune activation [22]. Recently, atypical chemokine receptors (ACKRs) have been described, which seem to bear a crucial impact on autoinflammatory CNS disorders such as multiple sclerosis (MS) or its animal model, experimental autoimmune encephalomyelitis (EAE) [23, 24]. Unlike other receptors, ACKRs do not seem to induce G protein signaling, but act as scavenger receptors [25]. As one of the four ACKRs described to date, ACKR3 (also known as CXCR7) binds C-X-C motif chemokine ligand (CXCL) 11 and CXCL12 [23, 26]. Its activation ultimately permits leukocyte entry into the CNS [27] and furthermore seems to mediate migration of activated microglia [23, 24], the latter strongly influencing multiple CNS diseases such as MS or Alzheimer’s disease, but also stroke or brain traumata, and even psychiatric diseases [28]. In animal models, ACKR3 was shown to be expressed within various brain regions, with an upregulation observable in case of inflammation, as for instance in EAE [29]. ACKR3 increases were furthermore found upon cytokine stimulation or induction of hypoxia in human astrocytes [30]. To date, only a single study addressed ACKR3 expression and inducibility in human brain endothelial cells [26]. We evaluated ACKR3 responses of human brain microvascular endothelial cells (HBMEC) upon exposure to bacterial lipopolysaccharide (LPS) and tumor necrosis factor-α (TNF-α), and furthermore used ACKR3 levels to depict the interactions of *Ureaplasma* spp. with native and LPS-primed HBMEC.

## Methods

### Bacterial strains and culture conditions

*U. urealyticum* serovar 8 (Uu8) and *U. parvum* serovar 3 (Up3) were acquired from the American Tissue Culture Collection (ATCC; Uu8: ATCC 27618, Up3: ATCC 27815). As described previously [31], *Ureaplasma* isolates were cultured in a liquid in-house medium (“broth”) containing 82% autoclaved pleuropneumonia-like organism medium (Becton, Dickinson & Company, Franklin Lakes, NJ, USA), 10% heat-inactivated horse serum, 1% urea, and 0.002% phenol red (all: Sigma-Aldrich, St. Louis, CA, USA). The medium was adjusted to pH 6.5 after passage through a 0.2-μm filter membrane (Sartorius, Goettingen, Germany). Using the ToxinSensor™ Endotoxin Detection System (GenScript, Piscataway, NJ, USA), we could verify an endotoxin level < 0.06 EU/ml broth. Serial 10-fold dilutions of the *Ureaplasma* cultures were incubated for 18–20 h to obtain titers of 1 × 10^9^–1 × 10^10^ color-changing units (CCU)/ml of viable organisms.

### Cell line and culture conditions

Non-immortalized HBMEC originating from adult human brain cortex were obtained from Cell Systems, Kirkland, WA, USA (ACBRI 376). HBMEC were grown to confluence in gelatin (Serva Electrophoresis, Heidelberg, Germany)-coated T-75 culture flasks (Greiner Bio-One, Frickenhausen, Germany). Cells were cultured in RPMI-1640 medium (Sigma-Aldrich), supplemented with 10% fetal calf serum (FCS) (Thermo Fisher Scientific, Waltham, MA, USA), 10% Nu-Serum (BD Biosciences, San Jose, CA, USA), 2 mM l-glutamine (Thermo Fisher), 1 mM sodium pyruvate (Thermo Fisher), 1% minimum essential medium non-essential amino acids (Thermo Fisher), 5 U/ml heparin (Biochrom, Berlin, Germany), and 0.3% endothelial cell growth supplement (Cell Systems). Cultures were kept in a humid atmosphere at 37 °C with 5% CO_2_. Confluent monolayers were washed with phosphate buffered saline (PBS) (Sigma-Aldrich), trypsinized (Sigma-Aldrich), and expanded into new culture flasks. After six passages, cells were harvested, centrifuged, resuspended in FCS + 10% dimethyl sulfoxide (Serva Electrophoresis), and immediately frozen at − 80 °C. Experiments were coherently conducted with recently thawed cells at passage 8. Preliminary experiments confirmed basic endothelial cell attributes of HBMEC such as a characteristic spindle-shaped growth pattern and expression of the endothelial marker CD31; furthermore, intercellular adhesion molecule 1 (ICAM-1) proved to be inducible in HBMEC (Additional file 1).

### Stimulation assays

For mRNA analysis and flow cytometry, HBMEC were transferred to gelatin-coated six-well culture plates (Greiner Bio-One) at a density of 2 × 10^5^ cells/well and grown to confluence for 48 h. Cells were washed, and 1 ml fresh growth medium was added per well. For immunocytochemistry, HBMEC were seeded on gelatin-coated 24-well culture plates (Thermo Fisher) at a density of 5 × 10^4^ cells/well and cultivated for 48 h. Cells were washed, and 0.5 ml fresh growth medium was added per well. Uu8 and Up3 suspensions were fourfold concentrated by centrifugation and resuspended in fresh broth and 10^9^–10^10^ CCU in 250 μl broth were added per ml of HBMEC medium. CCU were determined by 10-fold titration, as described previously [31], and corresponding genome equivalents were identified (Institute of Medical Microbiology and Hospital Hygiene, Duesseldorf, Germany). 10^9^–10^10^ CCU/ml equated to 5 × 10^7^–6 × 10^8^ copy numbers/ml. *Ureaplasma* concentrations used in this study corresponded with in vivo amniotic fluid levels in pregnancies with premature rupture of membranes < 37 weeks, which were reported to range from 4 × 10^2^ to 5 × 10^7^ copy numbers/ml in one study [32], while another described bacterial numbers between 1.9 × 10^7^ and 1.1 × 10^12^/ml [33]. Bacterial viability was verified by simultaneous culture on selective agar plates (medco Diagnostika GmbH, Ottobrunn, Germany). For negative controls in selected experiments, samples of *Ureaplasma* spp. were heat-killed at 60 °C for 15 min. LPS from *E. coli* serotype 055:B5 (Sigma-Aldrich) was added to HBMEC at a concentration of 100 ng/ml. TNF-α (Sigma-Aldrich) was used at a concentration of 10 ng/ml. Doses of LPS, TNF-α, and *Ureaplasma* spp. were determined by preliminary experiments (Additional file 2) analogous to previous approaches [26, 31, 34–36], testing 100, 500, 1000, and 2000 ng/ml LPS; 10, 40, and 100 ng/ml TNF-α; and 0.2, 0.3, 0.4, 0.5, and 1 × 10^9^–10^10^ CCU/ml *Ureaplasma* isolates. According to the results of preliminary time kinetic experiments (Additional file 2) with 2-, 4-, 6-, 20-, 24-, 30-, 36-, and 48-h incubation periods, exposure times of 4 and 30 h were chosen for mRNA analysis, while flow cytometry assays were performed after 24 and 48 h. Unstimulated HBMEC served as negative controls. In order to adjust for potential confounding effects of the in-house medium, HBMEC exposed to *Ureaplasma* isolates were compared to broth control in all experiments.

### RNA extraction and reverse transcriptase PCR (RT-PCR)

Total RNA was extracted using NucleoSpin® RNA Kit (Macherey-Nagel, Dueren, Germany) according to the manufacturer’s protocol. Total RNA was eluted in 60 μl nuclease-free H_2_O (Sigma-Aldrich), quantified using a Qubit® 2.0 Fluorometer (Thermo Fisher), and stored at − 80 °C until reverse transcription. For RT-PCR, 1 μg of total RNA was reverse transcribed using High-Capacity cDNA Reverse Transcription Kit (Thermo Fisher) according to the manufacturer’s instructions. First-strand cDNA was diluted 1:10 with deionized, nuclease-free H_2_O (Sigma-Aldrich) and stored at − 20 °C until analysis.

### Real-time quantitative RT-PCR (qRT-PCR)

For quantitative detection of ACKR3 mRNA, cDNA was analyzed in duplicates of 25 μl reaction mixture containing 12.5 μl iTaq™ Universal SYBR® Green Supermix (Bio-Rad Laboratories, Hercules, CA, USA), 0.5 μl deionized H_2_O, and 1 μl of a 10 μM solution of forward and reverse primer (gene symbol ACKR3, sequence accession # NM_020311.2, forward 5′-CGGAGGTCATTTGATTGCCC-3′, reverse 5′-AAGGAGAGCGTGTAGAGCAG-3′, amplicon length 176 bp, Sigma-Aldrich). PCRs were performed using an Applied Biosystems® 7500 Real-Time PCR System (Thermo Fisher). The two-step PCR protocol included an initial denaturation at 95 °C for 10 min and 40 cycles of 95 °C for 15 s and 60 °C for 1 min. A melt curve analysis at the end of every run verified single PCR products. Amplification was normalized to the reference gene HPRT1 (hypoxanthine phosphoribosyltransferase 1, sequence accession # NM_000194.2, forward 5′-CTGGCGTCGTGATTAGTG-3′, reverse 5′-AGTCCTGTCCATAATTAGTCC-3′, amplicon length 121 bp, Sigma-Aldrich). Mean fold changes in mRNA expression were calculated using the ΔΔC_T_ method [37]. Experiments were repeated five times (*n* = 5).

### RNA sequencing

Total RNA was extracted using NucleoSpin® RNA Kit (Macherey-Nagel) according to the manufacturer’s protocol, and samples were stored at − 80 °C until further processing. Experiments were repeated three times (*n* = 3). Library preparation was performed at the Core Unit Systems Medicine, University of Wuerzburg, Germany, according to the Illumina TruSeq stranded mRNA Sample Preparation Guide, using the Illumina TruSeq stranded mRNA Kit (both: Illumina, San Diego, CA, USA) with 700 ng of input RNA and 13 PCR cycles. Thirteen to fourteen libraries were pooled and sequenced on a NextSeq 500 (Illumina) with a read length of 75 nucleotides. A total of ~ 34–40 million raw reads per library were produced and processed using FastQC 0.11.5 [38] for assessing read quality, amount of duplicates, and presence of adapter sequences. The Illumina TruSeq adaptors were cleaved using cutadapt (version 1.14) [39]. Reads were additionally trimmed keeping a quality drop value below a mean of Q20. The processed sequences were mapped to the human genome using the short read aligner STAR (version 2.5.2b) [40] with genome and annotation files retrieved from GENCODE (version 25—March 2016 freeze, GRCh38). For all samples, the proportion of reads mapped to the human reference genome ranged between 76 and 90% in total. Sequences aligning to specific genes were quantified using bedtools subcommand intersect (version 2.15.0) [41]. Differentially expressed genes were identified with the help of DESeq2 (version 1.16.1) [42]. Only genes with a Benjamini-Hochberg corrected *p* value below 0.05 were classified as significantly differentially expressed. To compare different groups, reads per kilo base per million mapped reads (RPKM) were calculated for individual genes using DGEList and RPKM function from edgeR [43].

### Flow cytometry

Cells were harvested and incubated with 4 mg/ml Gamunex 10% (Grifols, Frankfurt, Germany) for Fc-receptor blocking. Cells were separated by centrifugation and stained with APC-H7-conjugated fixable viability dye (eBioScience, San Diego, CA, USA), a BV510-conjugated antibody to cluster of differentiation (CD) 31 (platelet endothelial cell adhesion molecule-1, BD Biosciences), and an APC-conjugated antibody to surface ACKR3 (BioLegend, San Diego, CA, USA). After centrifugation, cells were resuspended in PBS (Sigma-Aldrich) containing 1% human serum (Biochrom GmbH) and samples were read on a FACSCanto™ II flow cytometer (BD Biosciences), acquiring a minimum of 10,000 events to be analyzed with FACSDiva v6.1.3 software (BD Biosciences). Events were gated via forward and side scatter. A SSC-height versus FSC-width dot plot was used to exclude doublets, and cells were gated for viability-dye negative events. CD31 functioned as endothelial marker. Experiments were repeated five times (*n* = 5).

### Immunocytochemistry

After 24 h incubation, cells were rinsed twice with PBS (Lonza, Basel, Switzerland), fixed with 3.7% formaldehyde (AppliChem, Darmstadt, Germany), permeabilized with 0.1% Triton X-100 (Sigma-Aldrich) for 10 min, and blocked with 5% bovine serum albumin (Sigma-Aldrich) for 1 h at room temperature. An anti-ACKR3 antibody (1:2000, #PA3–069, Thermo Fisher) was incubated overnight at 4 °C. A corresponding secondary anti-rabbit-Cy3 antibody (1:400, Jackson ImmunoResearch, West Grove, PA, USA) was added for 1 h at room temperature. Nuclei were visualized with a DAPI staining (Thermo Fisher). Mowiol (Sigma-Aldrich) was added as an anti-fading agent. Images were taken using a Leica DMi8 inverted microscope (Leica Microsystems, Wetzlar, Germany) with fixed exposure times. Experiments were repeated three times (*n* = 3).

### Statistical analysis

PCR, RNA sequencing, and flow cytometry results were analyzed by a one-way ANOVA with Tukey’s multiple comparisons test using Prism® 6 software (GraphPad Software, San Diego, CA, USA). To account for normal distribution, data were transformed logarithmically using the equation LN(*x* + 0.1). Throughout this manuscript, multiplicity adjusted *p* values were calculated with log-transformed data, whereas untransformed data were employed for diagrams or calculation of *x*-fold change and standard deviation (SD). Differences with *p* < 0.05 were considered to be statistically significant. Data are shown as mean ± SD.

## Results

### Basal expression of ACKR3 mRNA in HBMEC

With cycle threshold values between 22.9 and 24.9 in native HBMEC, qRT-PCR results indicated relevant amounts of ACKR3 mRNA even in unstimulated HBMEC. Basal ACKR3 gene expression was confirmed by RNA sequencing, with ACKR3 RPKM ranging from 1.8 to 2.8 in unstimulated cells.

### ACKR3 responses of HBMEC upon stimulation with LPS, TNF-α, and *Ureaplasma* isolates

**Fig. 1:**
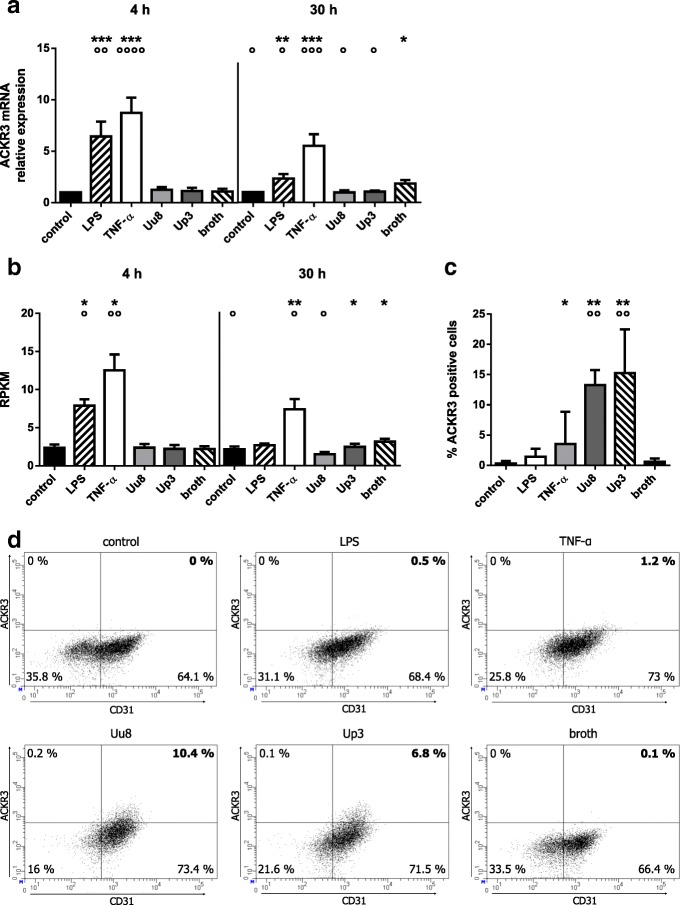
ACKR3 mRNA and protein expression in HBMEC after stimulation with LPS, TNF-α, or *Ureaplasma* spp. Levels of mRNA were assessed by qRT-PCR (**a**) and RNA sequencing (**b**). Flow cytometry was used to determine protein expression, events were gated for vital cells, with CD31 functioning as endothelial marker. For a stimulation period of 48 h, (**c**) shows the percentage of ACKR3- and CD31-positive cells, whereas dot plots of one representative experiment are given in (**d**). Data are presented as mean ± SD and significances are reported for comparisons vs. control and vs. broth (*p* values after logarithmic transformation, *p < 0.05, **p < 0.01, ***p < 0.001 vs. unstimulated control; °*p* < 0.05, °°*p* < 0.01, °°°*p* < 0.001, °°°°*p* < 0.0001 vs. broth)

Via qRT-PCR, we detected a significant increase of ACKR3 mRNA in HBMEC upon stimulation with *E. coli* LPS at 4 h (6.4-fold ±1.5, *p* = 0.0004, vs. control) and 30 h (2.3-fold ±0.4, *p* = 0.004) (Fig. 1a). TNF-α triggered an even more pronounced increase of ACKR3 mRNA (4 h: 8.7-fold ±1.5, *p* = 0.0001; 30 h: 5.5-fold ±1.1, *p* = 0.0004). RNA sequencing supported these data, documenting enhanced levels of ACKR3 mRNA upon LPS stimulation (4 h: 3.3-fold ± 0.4, *p* = 0.017) and TNF-α exposure (4 h: 5.3-fold ± 0.9, *p* = 0.011; 30 h: 3.4-fold ± 0.6, *p* = 0.006) (Fig. 1b). ACKR3 protein expression, on the contrary, was not influenced by LPS and only inconsistently increased by TNF-α (Fig. 1c).

Although exposure of HBMEC to Uu8 or Up3 did not significantly amplify ACKR3 mRNA in our setting (Fig. 1a, b), flow cytometry revealed a moderate induction of ACKR3 protein after 48 h of *Ureaplasma* stimulation (Uu8: 22.1-fold ± 4.1, *p* = 0.003; Up3: 25.4-fold ± 12.1, *p* = 0.002, vs. broth) (Fig. 1c, d). ACKR3 responses did not significantly differ between Uu8 and Up3. Heat-inactivated *Ureaplasma* spp. did not modulate ACKR3 levels (data not shown).

### ACKR3 response of HBMEC upon co-stimulation with LPS and *Ureaplasma* spp.

**Fig. 2:**
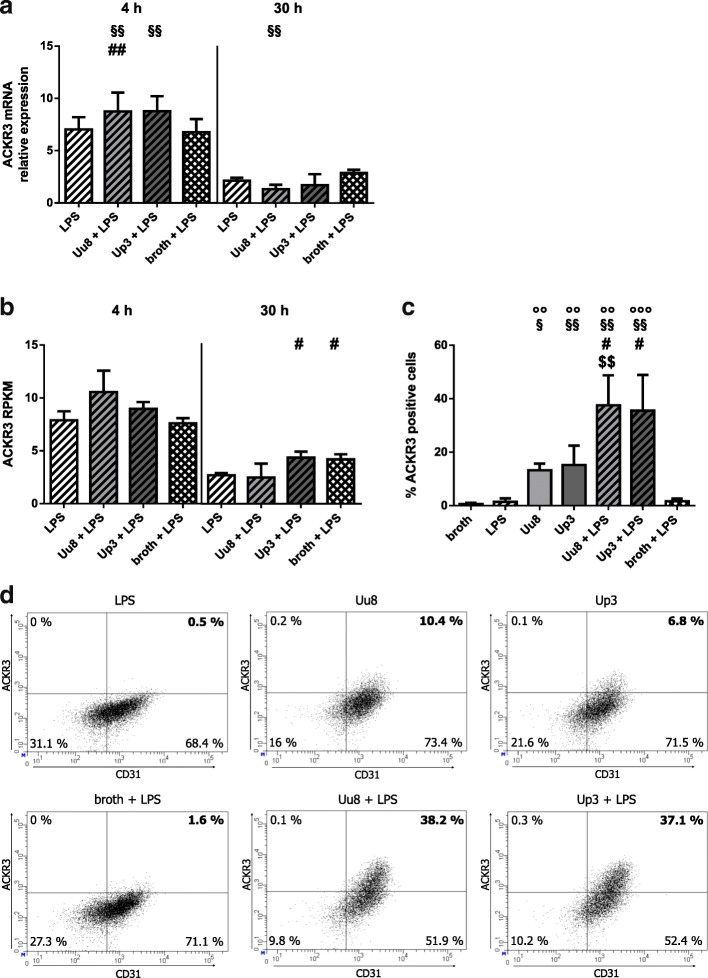
ACKR3 mRNA and protein expression in HBMEC after co-stimulation with LPS and *Ureaplasma* spp. ACKR3 mRNA expression was assessed using qRT-PCR (**a**) and RNA sequencing (**b**). Protein expression was determined by flow cytometry, with (**c**) representing the percentage of ACKR3- and CD31-positive HBMEC, and (**d**) depicting representative dot plots, both after a stimulation period of 48 h. Dot plots were gated for vital cells. Data are shown as mean ± SD (*p* values after logarithmic transformation, °°*p* < 0.01, °°°*p* < 0.001 vs. broth; #*p* < 0.05, ##*p* < 0.01 vs. LPS; §*p* < 0.05, §§*p* < 0.01 vs. broth + LPS; $$*p* < 0.01 vs. isolate alone)

In LPS-primed HBMEC co-incubated with *Ureaplasma* isolates for 4 h, we found an increase in LPS-induced ACKR3 mRNA, reaching statistical significance upon Uu8 exposure (1.2-fold ± 0.3, *p* = 0.007, vs. LPS) and borderline significance upon Up3 stimulation (1.2-fold ± 0.2, *p* = 0.052) (Fig. 2a). This amplifying effect remained significant if compared to broth (Uu8 + LPS: 1.3-fold ±0.3, *p* = 0.005; Up3 + LPS: 1.3-fold ±0.2, *p* = 0.001, vs. broth + LPS). Co-stimulation for 30 h did not significantly affect ACKR3 mRNA expression.

RNA sequencing revealed a non-significant trend towards higher ACKR3 mRNA levels following 4 h *Ureaplasma* exposure of LPS-primed cells (Fig. 2b).

As far as protein expression was concerned, we detected profoundly enhanced ACKR3 protein levels after 48 h of co-incubation with LPS and *Ureaplasma* isolates compared to stimulation with either one alone (Uu8 + LPS: 26.1-fold ± 7.8, *p* = 0.032, vs. LPS, and 2.8-fold ± 0.8, *p* = 0.006, vs. Uu8; Up3 + LPS: 24.7-fold ± 9.3, *p* = 0.038, vs. LPS, and 2.3-fold ± 0.9, *p* = 0.104, vs. Up3) (Fig. 2c, d). Again, both isolates did not significantly differ in their stimulatory capacity.

### Immunocytochemistry

**Fig. 3:**
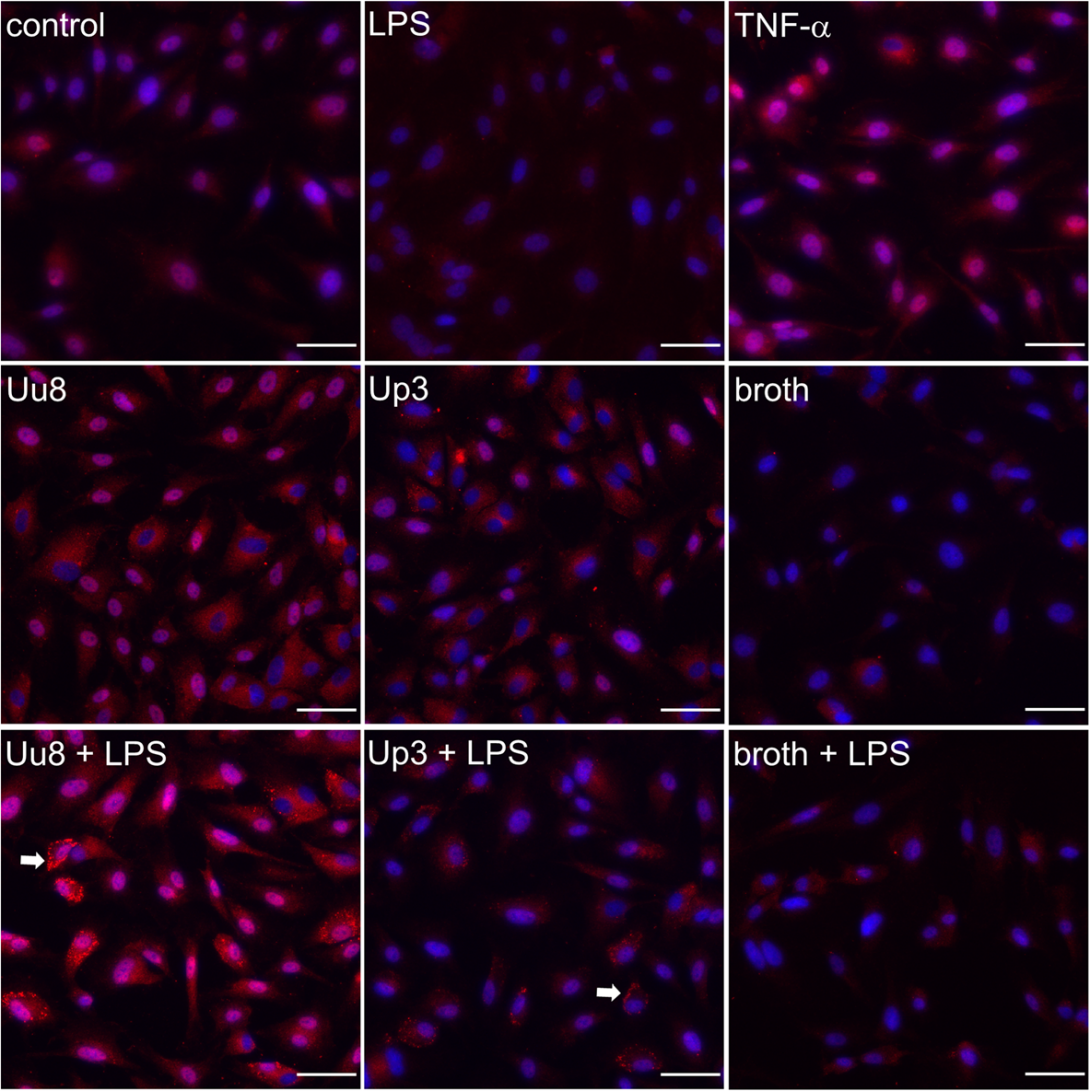
ACKR3 immunocytochemistry in native and stimulated HBMEC. Representative images of basal and inflammation-induced ACKR3 (red) expression in HBMEC. Nuclei were visualized with DAPI (blue). ACKR3-positive granules in LPS-primed HBMEC additionally incubated with *Ureaplasma* spp. are indicated (arrow). Scale bar = 50 μm

Native HBMEC as well as those stimulated with LPS or TNF-α exhibited a faint and homogenous cytoplasmic staining for ACKR3 (Fig. 3). Incubation with *Ureaplasma* spp. enhanced the cytoplasmic signal. Upon *Ureaplasma*-stimulation of LPS-primed HBMEC, we additionally observed intense submembranous granules.

### CXCL11 and CXCL12 responses of HBMEC upon stimulation

**Fig. 4:**
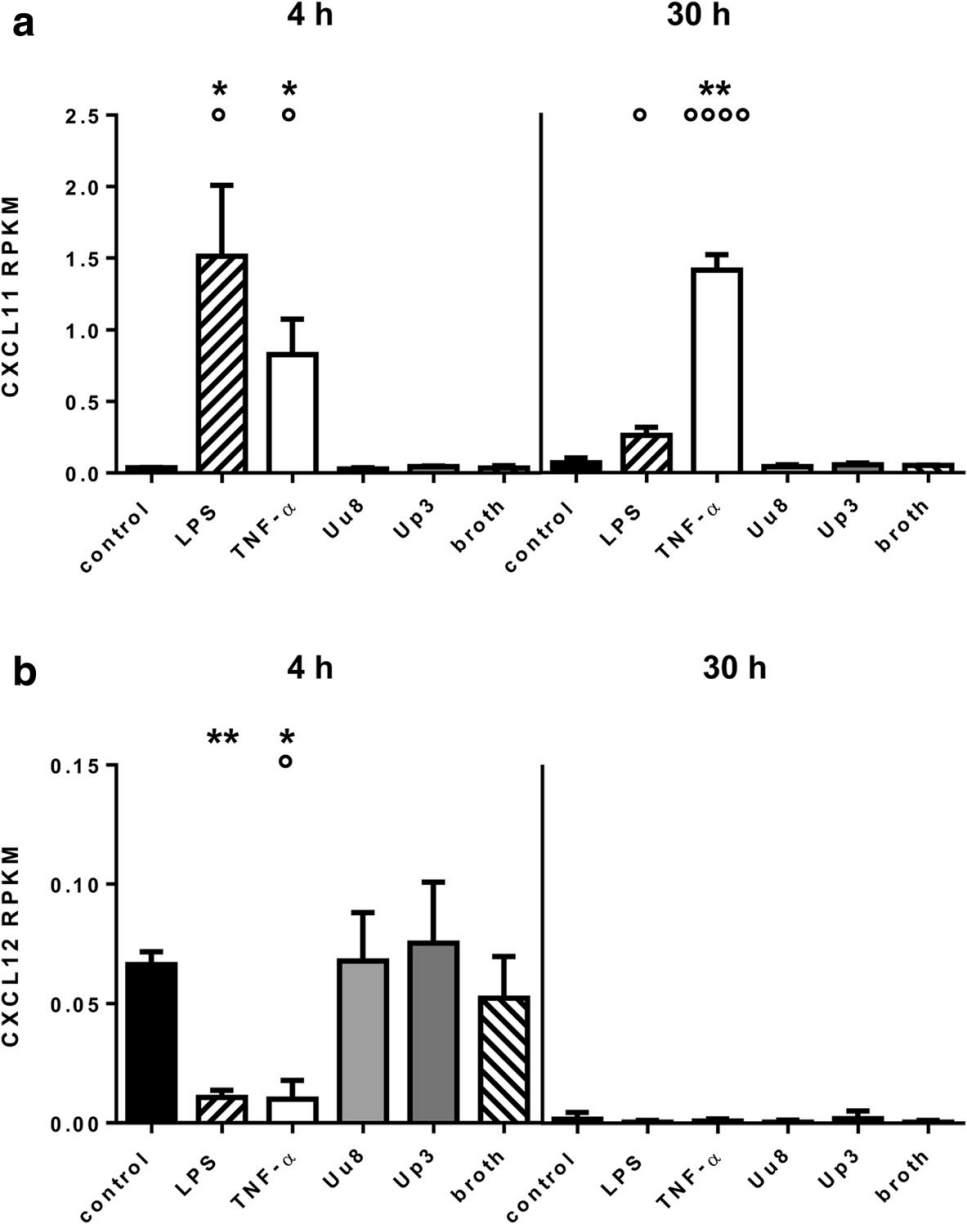
CXCL11 and CXCL12 mRNA levels in HBMEC after stimulation with LPS, TNF-α, or *Ureaplasma* spp. RNA sequencing data demonstrate CXCL11 (**a**) and CXCL12 (**b**) mRNA responses upon stimulation with LPS and TNF-α. Values represent mean RPKM ± SD (*p* values after logarithmic transformation, *p < 0.05, **p < 0.01 vs. unstimulated control; °*p* < 0.05, °°°°*p* < 0.0001 vs. broth)

Having demonstrated a stimulus-induced increase of ACKR3 mRNA expression in HBMEC, we examined the impact of LPS, TNF-α, Uu8, and Up3 on the receptor’s ligands CXCL11 and CXCL12 (Fig. 4). Using RNA sequencing data, we assessed enhanced levels of CXCL11 mRNA upon stimulation with LPS for 4 h (42.4-fold ± 13.8, *p* = 0.014, vs. control) and TNF-α for 4 h and 30 h (4 h: 23.1-fold ± 6.9, *p* = 0.022, 30 h: 20.0-fold ± 1.5, *p* = 0.006). CXCL12 mRNA, on the other hand, was significantly reduced after a 4 h exposure of HBMEC to LPS (0.161-fold ± 0.044, *p* = 0.006) or TNF-α (0.150-fold ± 0.119, *p* = 0.024), whereas no significant effects of a stimulation remained after 30 h.

Exposure to *Ureaplasma* spp. did not influence CXCL11 or CXCL12 mRNA levels both in native and LPS-primed HBMEC (Fig. 4).

## Discussion

A compromised integrity of the blood-brain barrier (BBB) is a common pathological feature in numerous inflammatory disorders of the CNS [44, 45]. Recently, ACKR3 has been recognized as a contributor to BBB impairment by permitting passage of inflammatory cells into the CNS in the event of inflammation [27].

The present study is the first to analyze pro-inflammatory influences of *Ureaplasma* spp. as well as LPS and TNF-α on HBMEC, which constitute a major component of the BBB [45]. Our data reveal that all given stimuli are able to induce ACKR3 responses in HBMEC. These results (i) suggest a novel role of ACKR3 not only in autoimmune neuroinflammation, but also in CNS infections; (ii) introduce an in vitro model of *Ureaplasma* meningitis; and (iii) for the first time indicate a pro-inflammatory capacity of *Ureaplasma* spp. in HBMEC.

The current data demonstrate basal ACKR3 mRNA expression in unstimulated HBMEC. These results are in accordance with the only previous study addressing ACKR3 expression in HBMEC [26]. In addition, we found a significant increase in ACKR3 mRNA upon stimulation not only with TNF-α, as described in one previous study [26], but also with LPS. While TNF-α is an endogenous pro-inflammatory cytokine particularly involved in autoimmune disorders [46, 47], LPS, as a component of bacterial membranes, is widely used to experimentally mimic gram-negative bacterial infections. Our data on LPS-induced ACKR3 amplification therefore propose an involvement of ACKR3 not only in autoimmune neuroinflammatory disorders, but also in infection-associated CNS diseases. These results are in line with previous studies describing LPS-inducible ACKR3 expression in choroid endothelial cells [48], a colorectal carcinoma cell line [49], and pulmonary tissue [50] and may provide novel insights into pathophysiological processes in meningitis.

This study is the first to address the interactions of viable *Ureaplasma* isolates with cells constituting the BBB. While pro-inflammatory cytokine responses to *Ureaplasma* colonization or infection were described for chorioamnion [51] and lung [52], no such correlation was made for the CNS to date. By demonstrating an ability of *Ureaplasma* spp. to induce ACKR3 in HBMEC, our data provide evidence of a pro-inflammatory capacity of *Ureaplasma* spp. in brain endothelial cells. Several case reports identified *Ureaplasma* spp. as causative in neonatal meningitis [14, 15], and some authors furthermore described a higher risk for intraventricular hemorrhage (IVH) or cerebral palsy (CP) in *Ureaplasma*-colonized preterm infants [2, 53, 54]. Taking together these observations and our in vitro findings, the clinical relevance of *Ureaplasma* spp. in inflammatory CNS disorders may be much higher than contemplated to date. *Ureaplasma*-driven neuroinflammation may be mediated by ACKR3, potentially in terms of a consecutive BBB breakdown, ultimately allowing the influx of inflammatory cells into the CNS.

Flow cytometry and immunocytochemistry data from HBMEC co-stimulated with LPS and *Ureaplasma* spp. indicate impairments of BBB integrity particularly in the event of co-infection with *Ureaplasma* spp. and a second pathogen. While evoking only moderate ACKR3 responses themselves, *Ureaplasma* spp. caused an over-proportional ACKR3 increase in HBMEC if combined with LPS. These results are in line with in vitro studies in human monocytes, describing an aggravation of LPS-induced inflammatory responses by *Ureaplasma* spp. [31, 55, 56]. Furthermore, clinical data revealed a more intense intrauterine inflammation in polymicrobial chorioamnionitis with *Mycoplasma* or *Ureaplasma* spp. and other bacteria compared to amniotic infection with other bacteria alone [57]. Together, this may indicate an immunomodulatory capacity of *Ureaplasma* spp., which is likely to be of particular clinical relevance. Polymicrobial colonization and co-infections are a common problem particularly in an intensive care setting, and *Ureaplasma* spp. may predispose for invasive CNS infections with other pathogens. However, clinical data addressing this topic are pending.

The exact mechanism of ACKR3 induction by *Ureaplasma* spp. is yet to be determined. However, results from a knock-out experiment in a colorectal carcinoma cell line suggest that LPS-induced increase in ACKR3 expression might be mediated by Toll-like receptor (TLR) 4 [49]. Considering that TLR signaling is crucial in mediating pro-inflammatory effects evoked by *Ureaplasma* spp. [58, 59], TLRs might be involved in the pathway of *Ureaplasma*-driven ACKR3 induction as well. In earlier studies, we could demonstrate an *Ureaplasma*-induced TLR2 mRNA increase in neonatal and adult monocytes [31]. Opposed to TLR4, generally being a potent activator of LPS-induced immune responses, TLR2 primarily mediates bacterial lipopeptide recognition [60]. Differences in TLR engagement may therefore be a potential explanation for the diverging effects of LPS and *Ureaplasma* spp. on mRNA and protein levels observed in this study. These aspects need to be addressed in further studies.

ACKR3 acts as a receptor for chemokines CXCL11 and CXCL12 [23, 26] and is known to scavenge its ligands to some extent [25, 61]. CXCL12 restricts leukocyte entry into the CNS [62] and is therefore relevant in limitation of inflammation. Animal studies indicate a beneficial role for CXCL12 in post-ischemia white matter injury [63], whereas antagonist studies provide evidence that an internalization of CXCL12 by ACKR3 and the resulting decline of CXCL12 allow consecutive inflammatory parenchymal infiltration [27]. Loss of CXCL12 has been observed in MS [64], but may be relevant in other inflammatory morbidities as well. In accordance with these observations, we could demonstrate reduced CXCL12 mRNA levels in HBMEC upon exposure to LPS and TNF-α. The second receptor ligand CXCL11 and its interactions with ACKR3 are less well characterized, but CXCL11 also seems to relevantly contribute to EAE [65] and is adjudged a role in chemotaxis of mononuclear cells [65, 66]. Inverse to CXCL12, we found an increase of CXCL11 in response to LPS and TNF-α stimulation of HBMEC. In line with previous studies [27, 65, 66], these results may provide evidence for CXCL12 loss and CXCL11 increase being the causal link between ACKR3 elevation and consecutive neuroinflammation. Furthermore, ACKR3 mediates microglial chemotaxis, relevant in several neuroinflammatory morbidities [28], by activation of extracellular signal-regulated kinases (ERK) 1/2 [24]. This mechanism offers yet another possible explanation for the relevance of ACKR3 in inflammatory diseases of the CNS.

Moreover, given the abovementioned protective role of CXCL12 in white matter integrity [63], our results may imply a relevance of ACKR3 in other diseases potentially associated with *Ureaplasma* spp., such as IVH or CP. AKCR3 might be a missing link between pathogen and morbidity, an aspect that should be addressed in future studies.

The strength of this study relates to the use of two different, viable *Ureaplasma* serovars and the assessment of pro-inflammatory responses at the level of mRNA synthesis and protein expression as well as visualization of ACKR3 expression via immunocytochemistry.

One potential limitation of this study is the use of a commercially available single donor cell line. Although commonly used and widely accepted, these cell lines bear a certain risk of not being fully representative. Furthermore, this study was conducted with an adult cell line. Considering the risk cohort of preterm and term neonates, a freshly isolated cell line, ideally of neonatal origin, is desirable and should be employed in further studies. Moreover, HBMEC used in this study originated from cortex tissue. Although a recent study found an equal distribution of typical BBB markers in parenchymal and meningeal vessels [67], cortical HBMEC may still differ from the meningeal endothelial cells also contributing to the BBB, and results should not be unconditionally transferred from one to the other.

## Conclusions

While BBB breakdown has generally been acknowledged as being a pathophysiological basis of neuroinflammatory diseases, the impact of ACKR3 has only just begun to be appreciated. Whereas, to date, ACKR3 has been adjudged a key role primarily in autoimmune neuroinflammatory disorders, our results suggest its additional involvement in infectious diseases of the CNS. *Ureaplasma* spp. and other pathogens might be able to initiate a cascade beginning with ACKR3 induction, proceeding to BBB breakdown, and ultimately cumulating in inflammatory brain injury. This may be mediated by CXCL12 loss and CXCL11 increase, as some of our results suggest.

This study is the first to introduce an in vitro model of *Ureaplasma* meningitis. Our findings of *Ureaplasma*-induced ACKR3 expression demonstrate a pro-inflammatory capacity of *Ureaplasma* spp. in HBMEC. Data from co-stimulated HBMEC indicate an immunomodulatory capacity of *Ureaplasma* spp. in the event of co-infection, which may be of particular clinical relevance.

## Additional files

Additional file 1: HBMEC fulfilled basic endothelial cell characteristics. CD31 protein was detectable in native HBMEC, as outlined in a representative flow cytometry histogram (A). QRT-PCR results furthermore demonstrated an inducibility of ICAM-1 in HBMEC by LPS or TNF-α (B), in this case after a 4-h stimulation period. Relative quantifications are shown as mean ± SD (*p* values after logarithmic transformation, ****p < 0.0001 vs. control; *n* = 5). (TIF 186 kb)

Additional file 2: Preliminary experiments with HBMEC. A dose dependent induction of ICAM-1 mRNA in HBMEC was revealed by qRT-PCR. LPS-evoked response peaked at 100 ng/ml (A), whereas TNF-α doses exceeding 10 ng/ml did not result in relevant further mRNA increase (B). Time kinetic experiments (C) showed highest ICAM-1 mRNA levels after a 4 h stimulation period, in this case with LPS 100 ng/ml. Data (A-C) are shown as relative quantifications resulting from four experiments. (TIF 300 kb)

## Abbreviations

ACKR3: Atypical chemokine receptor 3
BBB: Blood-brain barrier
CCU: Color-changing units
CD: Cluster of differentiation
CNS: Central nervous system
CP: Cerebral palsy
CXCL: C-X-C motif chemokine ligand
E.: *Escherichia*
EAE: Experimental autoimmune encephalomyelitis
FCS: Fetal calf serum
HBMEC: Human brain microvascular endothelial cells
HPRT1: Hypoxanthine phosphoribosyltransferase 1
ICAM-1: Intercellular adhesion molecule 1
IVH: Intraventricular hemorrhage
LPS: Lipopolysaccharide
MS: Multiple sclerosis
qRT-PCR: Real-time quantitative reverse transcriptase polymerase chain reaction
RPKM: Reads per kilo base per million mapped reads
SD: Standard deviation
spp.: Species
TLR: Toll-like receptor
TNF-α: Tumor necrosis factor-α
U.: *Ureaplasma*
Up3: *Ureaplasma parvum* serovar 3
Uu8: *Ureaplasma urealyticum* serovar 8
