# Supplementary figures and images for: Novel insights into neuroinflammation: bacterial lipopolysaccharide, tumor necrosis factor α, and Ureaplasma species differentially modulate atypical chemokine receptor 3 responses in human brain microvascular endothelial cells
Source: J Neuroinflammation. 2018 May 23;15:156. doi: 10.1186/s12974-018-1170-0 (PMC5966865; doi:10.1186/s12974-018-1170-0)

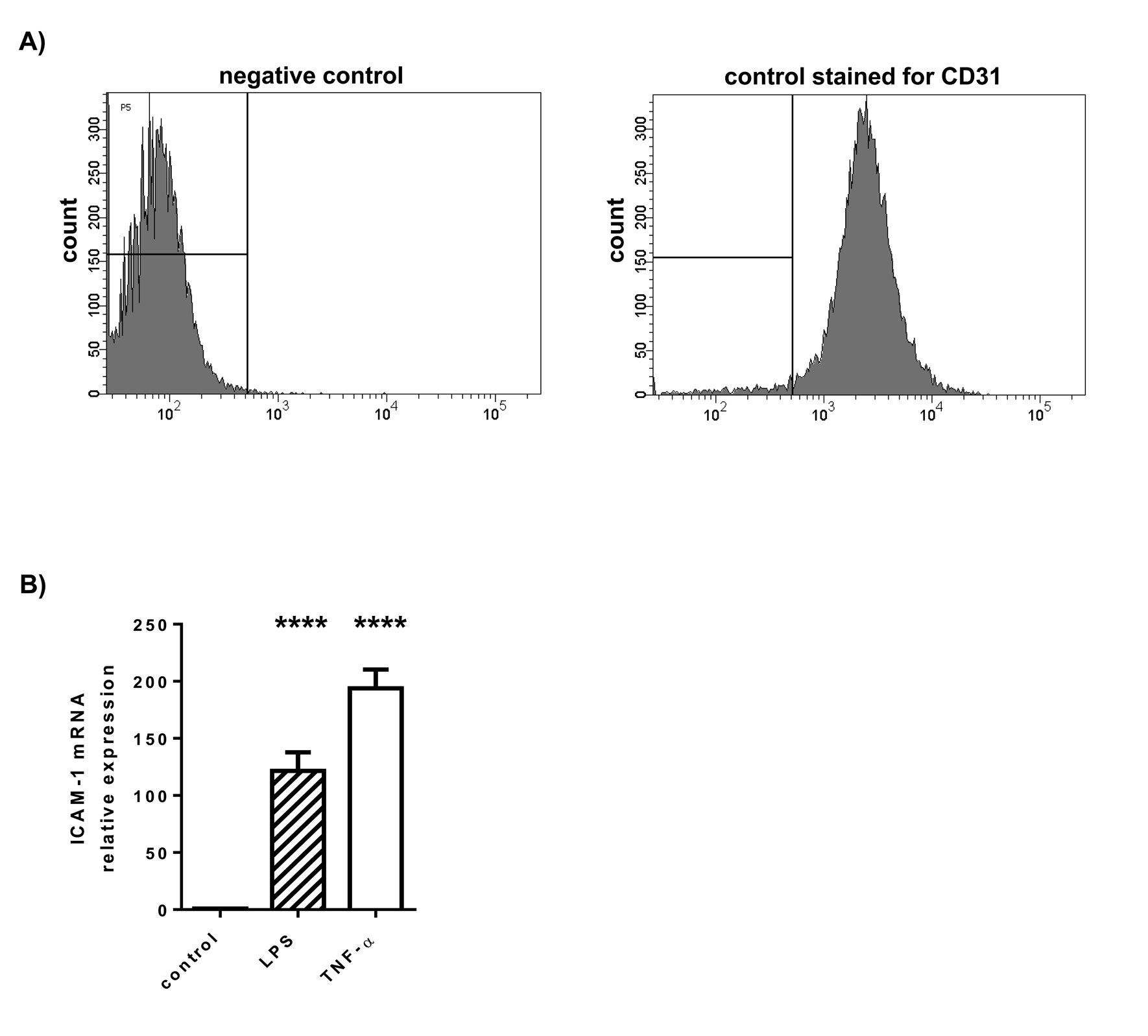

Supplement: Supplementary file 1 — HBMEC fulfilled basic endothelial cell characteristics. CD31 protein was detectable in native HBMEC, as outlined in a representative flow cytometry histogram (A). QRT-PCR results furthermore demonstrated an inducibility of ICAM-1 in HBMEC by LPS or TNF-α (B), in this case after a 4-h stimulation period. Relative quantifications are shown as mean ± SD (p values after logarithmic transformation, ****p < 0.0001 vs. control; n = 5). (TIF 186 kb) [file 12974_2018_1170_MOESM1_ESM.tif]

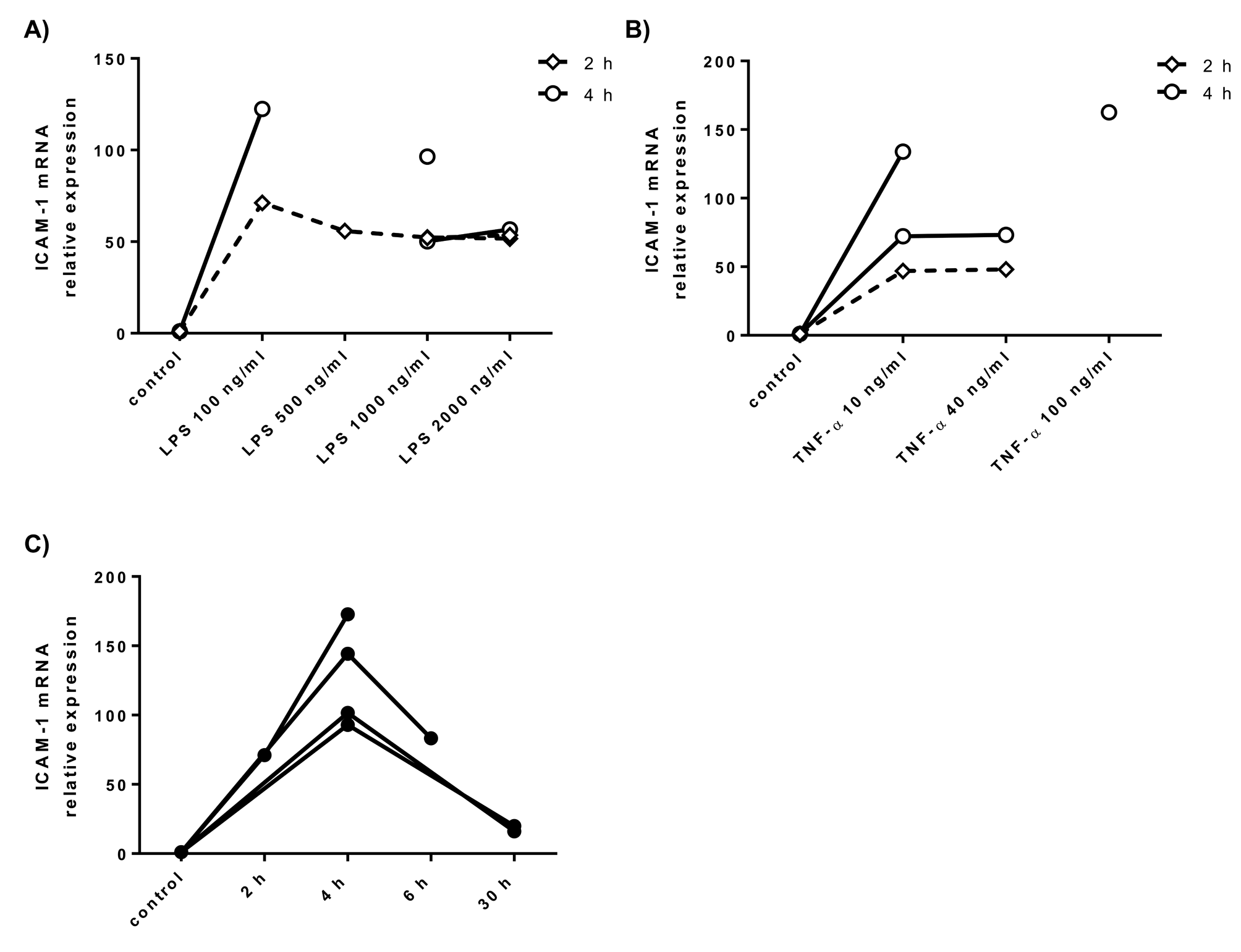

Supplement: Supplementary file 2 — Preliminary experiments with HBMEC. A dose dependent induction of ICAM-1 mRNA in HBMEC was revealed by qRT-PCR. LPS-evoked response peaked at 100 ng/ml (A), whereas TNF-α doses exceeding 10 ng/ml did not result in relevant further mRNA increase (B). Time kinetic experiments (C) showed highest ICAM-1 mRNA levels after a 4 h stimulation period, in this case with LPS 100 ng/ml. Data (A-C) are shown as relative quantifications resulting from four experiments. (TIF 300 kb) [file 12974_2018_1170_MOESM2_ESM.tif]
